# Probing the Small, Medium and Large Amplitude Rheological Properties of Cherry Jell-O^®^ as a Model System for Edible Gels

**DOI:** 10.3390/gels12040295

**Published:** 2026-04-01

**Authors:** Ozge Ata, Gamze Yazar, Harrison Helmick, Elise Whitley, Sebnem Tavman, Jozef L. Kokini

**Affiliations:** 1 Food Science Department, Purdue University, 745 Agriculture Mall Dr., West Lafayette, IN 47907, USA; oata@purdue.edu (O.A.); harrison.helmick@roquette.com (H.H.); ewhitle@purdue.edu (E.W.); 2 Department of Food Engineering, Faculty of Engineering, Ege University, 35100 Izmir, Türkiye; sebnem.tavman@ege.edu.tr; 3 Department of Animal, Veterinary and Food Sciences, University of Idaho, 875 Perimeter Dr. MS 2312, Moscow, ID 83844, USA; gamzey@uidaho.edu; 4 Roquette America Inc., Geneva, IL 60134, USA

**Keywords:** Jell-O^®^, gelatin, SAOS, MAOS, LAOS, FTIR

## Abstract

This study investigated the linear and nonlinear viscoelastic properties of cherry Jell-O^®^ samples through oscillatory shear methods including small-amplitude (SAOS), medium-amplitude (MAOS), and large-amplitude (LAOS) experiments. Cherry Jell-O^®^ showed solid-like gel behavior (tanδ < 1) up to γ_0_:160%. The sample transitioned into nonlinear behavior above γ_cri_: 16% and was classified as type III (weak strain overshoot). Chebyshev coefficients revealed that the samples exhibited strain-stiffening (e_3_/e_1_ > 0) and shear-thickening (v_3_/v_1_ > 0) intracycle behavior in the nonlinear region. Both elastic and viscous Lissajous–Bowditch curves showed distortions from elliptical trajectories in the nonlinear region. FTIR spectra showed LAOS deformation-induced structural changes, particularly in the Amide I and Amide II regions. Tanδ decreased below 1 upon the removal of the LAOS deformation. These findings showed that although LAOS deformation induced molecular changes in the cherry Jell-O^®^ samples, their elasticity was largely preserved by a strong, resilient network.

## 1. Introduction

Hydrocolloids play a crucial role in determining the functional characteristics and overall quality of food products. Formulated foods often contain blends of hydrocolloids that contribute to structural properties [1]. These hydrocolloid gels typically exhibit a wide range of behaviors, ranging from liquid-like to solid-like, depending on their molecular and compositional properties. Their liquid-like characteristics primarily arise from the high-water content of the gel, which typically exceeds 80%. In contrast, their solid-like behavior results from the formation of a three-dimensional network structure, reflected by a finite elastic modulus [2]. Additionally, some hydrocolloids, such as gelatin, agar, carrageenan, alginate, pectin, gellan gum, etc., can undergo reversible transitions between sol and gel states in response to temperature changes and shear history. This behavior is governed by covalent and non-covalent molecular interactions, including hydrogen bonding and hydrophobic forces [3]. Owing to these properties, hydrocolloids are widely used as structure-forming gelling agents in various food formulations.

Gelatin is a denatured form of collagen present in connective tissues such as bones and skin. Unlike collagen, gelatin is highly hydrophilic and is widely used in food and pharmaceutical products as a thickening, stabilizing, foaming, and gelling agent [4]. Gelatin exhibits thermo-reversible behavior, allowing it to transition between gel and sol states in response to temperature variations. The gelation of gelatin is governed by a reverse coil-to-helix transition that occurs upon cooling below 30 °C. During cooling, some polymer chains interact and partially align into collagen-like triple helical structures, ultimately resulting in gel network formation [5,6]. As a result of gelation, a firm and elastic semisolid colloidal gel is formed, which makes gelatin a desirable gelling agent for many gummy and jelly dessert products [7]. Gelatin-based gels have been widely studied for food applications in recent years, including meat preservation [8], food packaging [9], encapsulation and controlled release of scent molecules [10], artificial beef tendons [11], and antidiabetic peptides [12]. Jell-O^®^ is the most widely used commercial product that uses gelatin, an animal-derived protein hydrocolloid, and can be considered a practical example of protein-based hydrocolloid gel. In addition to gelatin, Jell-O^®^ contains sugar or other sweeteners, acidulants (such as adipic and fumaric acids, which help control pH), buffering agents (such as disodium phosphate and sodium citrate), artificial flavors, and colorants. These components contribute to its taste, color, pH stability, and gel strength. Jell-O^®^ is available in various flavors such as cherry, blackberry, blueberry, strawberry, raspberry, lemon, and orange, most of which are well accepted by consumers.

Several studies have investigated the stability and strength of gelatin-based gels in relation to co-molecules [13], physical treatments [14], and crosslinking methods [15], contributing to the design of gel-based food products. SAOS, MAOS, and LAOS tests provide an in-depth understanding of gel behavior across both the linear and nonlinear regions and are commonly used to evaluate gel stability and strength. The rheological behavior of gelatin hydrogels has been investigated using these tests [16,17,18]. The stability and strength of Jell-O^®^ have not been investigated comprehensively using SAOS, MAOS, and LAOS tests. This study is the first to characterize the linear and nonlinear viscoelastic properties of Jell-O^®^ and to examine the associated structural changes using FTIR. This study aims to use Jell-O^®^ as a model gel system to better understand the viscoelastic behavior of gelatin-based gels under deformations ranging from small to large. Since Jell-O^®^ is one of the most well-known commercial gel products, this study provides valuable insights into how to optimize gel-based food formulations.

## 2. Results and Discussion

### 2.1. Linear Viscoelastic Properties of Cherry Jell-O^®^

**Figure 1:**
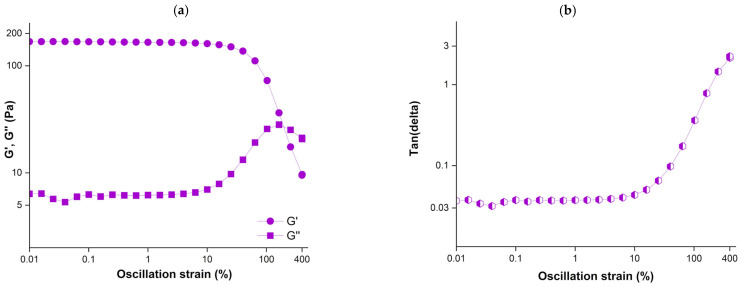
(**a**) Dependence of G′ and G″ moduli of cherry Jell-O^®^ versus strain (%) and (**b**) tanδ values versus strain (%) at 20 °C (γ_0_: 0.01–400%, ω: 10 rad/s).

Figure 1a shows the strain sweep curves for the samples. The transition to the nonlinear region began after a critical strain (γ_cri_) of 16% was reached, and the crossover between the storage modulus (G′) and the loss modulus (G″) occurred at approximately 160% strain. The mechanism behind the long transition zone between the critical strain and crossover strain is attributed to the presence of strong covalent bonds and interplay between the hydrogen bonds, which allowed the network to maintain its integrity and resist deformation, as commonly observed in gel systems [19,20]. After the crossover, hydrogen bonds and other relatively weak physical interactions were progressively disrupted, resulting in a fluid-like response.

G′ and G″ of the samples were approximately 168 Pa and 6 Pa, respectively, within the linear viscoelastic region. G′ remained higher than G″ throughout this region, indicating solid-like gel behavior, as expected. The tanδ values of the samples remained below 1 up to a strain amplitude (γ_0_) of approximately 160% (Figure 1b), indicating their elastic nature. However, tanδ values began to increase beyond γ_0_:160%, suggesting that the gel was affected by high strains and had lost its elastic structure. Mehdi-Sefiani et al. [16] reported a similar γ_cri_ value of 14.8% for a Type A gelatin hydrogel. The same study also showed that γ_cri_ increased up to 99.9% in the presence of tetracycline. This work further reported tanδ values ranged from 0.02 to 0.09, depending on the processing temperature. In the absence of tetracycline, tanδ values were not significantly affected by temperature and remained constant at approximately 0.03 [16], in agreement with our findings. Varela et al. [13] investigated the effect of honey incorporation into gelatin gels and reported that honey surprisingly enhanced their solid-like behavior, with G′ values increasing from 54.64 to 173.37 Pa, which are comparable to those observed in our study.

Nogami et al. [19] examined how various polymers, including hydroxypropyl methylcellulose (HPMC), hydroxypropyl methylcellulose acetate succinate, hydroxypropyl methylcellulose phthalate (HPMCP), and methacrylic acid–ethyl acrylate copolymer (Eudragit^®^), affect the rheological behavior of gelatin gels. They reported that all gelatin gels exhibited consistently low and stable tanδ values up to 100% strain, as observed in cherry Jell-O^®^ samples tested in this work (Figure 1b), followed by a sudden increase beyond this point. However, gelatin gels with HPMCP showed a more stable structure compared to those prepared with the other polymers. On the other hand, Tran Vo et al. [21] reported a sharp increase in tanδ values beyond their critical strains (γ_0_ ≈ 1%) in calcium–pectinate hydrogels prepared with different pectin concentrations, along with a loss of solid-like behavior after γ_0_: 10%. It can be concluded that the rheological properties of hydrocolloid-based gels can vary depending on the incorporation of crosslinkers and other components into the gel matrix, as well as adjusting the temperature.

### 2.2. Nonlinear Viscoelastic Properties

#### 2.2.1. MAOS Behavior

The medium amplitude oscillatory shear (MAOS) is described as the intermediate region between SAOS and LAOS and has attracted particular interest to better investigate the nonlinear rheological behavior of materials. In the MAOS region, the intrinsic nonlinear behavior can be probed without inducing significant structural disruption as compared to the LAOS region [22]. The first (1st) and third (3rd) harmonics of a material’s rheological response have been shown to characterize its medium amplitude oscillatory shear (MAOS) behavior [23]. Particularly, the intensity of the third harmonic is strongly associated with the underlying polymer structure, resulting in characteristic rheological signatures. The intensity of the third harmonic systematically captures the initial nonlinearities associated with the material’s nonlinear behavior [22,24]. As a result, the onset of the MAOS region of the material is characterized by the ratio of the third (I_3_) to the first (I_1_) harmonic intensity as a function of strain amplitude [23]. In other studies, it has been shown that the I_3_/I_1_ intensity ratio increases systematically within the MAOS region, and the deviation from this behavior defines the onset of the LAOS region [22,23,25].

**Figure 2:**
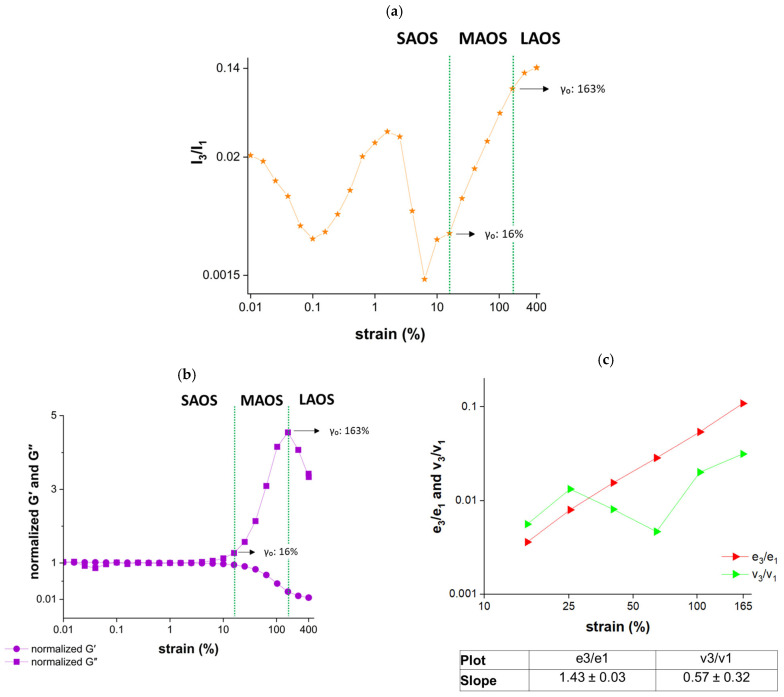
MAOS properties of cherry Jell-O^®^ samples. (**a**) MAOS map; (**b**) normalized G′ and G″; (**c**) the logarithmic change in the absolute values of e_3_/e_1_ and v_3_/v_1_ in the MAOS region.

According to the I_3_/I_1_ ratio shown in Figure 2a, the lower-strain MAOS boundary of cherry Jell-O^®^ was 16%, which is consistent with the critical strain value (γ_cri_:16%) calculated through the deviation in the G′ and G″ values during strain sweeps. The upper-strain MAOS boundary was determined to be 163%, beyond which the samples are in the LAOS region (Figure 2a).

As shown in Figure 2b, G″ exceeded G′ within the MAOS region. According to the nonlinear material behavior classification proposed by Hyun et al. [26], cherry Jell-O^®^ samples demonstrated type III nonlinear behavior (weak strain overshoot) as reflected by the decreasing G′ values and the parabolic trend in G″, where G″ first increased and then decreased (Figure 2b). At moderate strains, the gelatin network resisted deformation due to hydrogen bonds and hydrophobic interactions, resulting in a G″ overshoot. As the material transitioned into the LAOS region, the network was progressively disrupted. Then, the polymer chains began to align with the flow direction, resulting in a gradual decrease in G″ (Figure 2b).

Type III nonlinear behavior was also reported for gelatin/chitosan hydrogels [27], *Ficus pumila* polysaccharide-based wheat starch gels [28], and soy protein isolate emulsion gels [29]. The elastic coefficient (e_3_/e_1_) increased with strain, as shown in Figure 2c, indicating a progressive reduction in elastic nonlinearity with increasing deformation. In contrast, the viscous coefficient (v_3_/v_1_) showed an initial increase, followed by a decrease and then an increase again, suggesting strain-induced structural rearrangements. The higher slope of e_3_/e_1_ (1.43 ± 0.03) is the result of decaying elastic effects at large strains. On the other hand, the lower and fluctuating slope of v_3_/v_1_ (0.57 ± 0.32) can be associated with the break-up of bonds within the network, followed by their partial reformation as the structure reorganizes under deformation. At higher strains, the continued increase in v_3_/v_1_ suggested a more persistent breakdown of the network structure. This finding is also consistent with the overshoot observed in G″ (Figure 2b).

#### 2.2.2. LAOS Behavior

**Figure 3:**
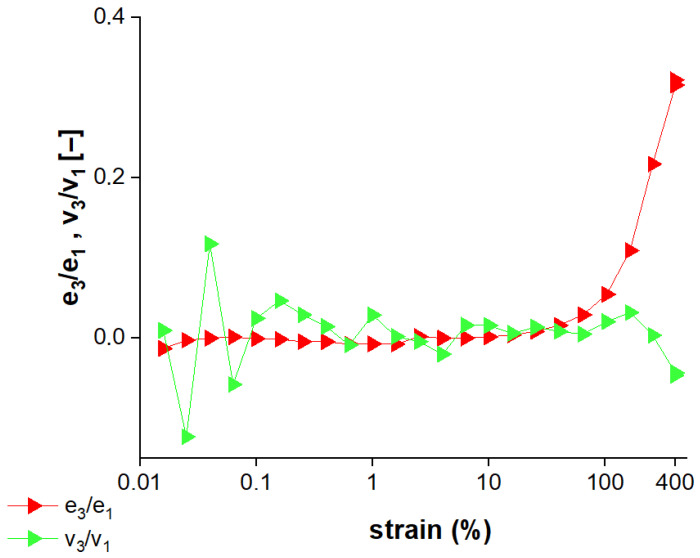
The variation in nonlinear coefficients (e_3_/e_1_, v_3_/v_1_) of cherry Jell-O^®^ with respect to strain (%).

Figure 3 shows the nonlinear Chebyshev coefficients (e_3_/e_1_ and v_3_/v_1_) used to evaluate the intracycle elastic and viscous nonlinearities, which indicate the onset of nonlinear viscoelastic behavior [30]. The elastic coefficient (e_3_/e_1_) remained very close to zero, indicating a predominantly linear elastic response up to γ_0_: 16–25%. On the other hand, the viscous coefficient (v_3_/v_1_) exhibited fluctuations, which appear to be due to experimental error, especially at very low strain amplitudes (γ_0_ ≤ 1.5%). As strain increased beyond 25%, both e_3_/e_1_ and v_3_/v_1_ began to deviate from zero, indicating the onset of nonlinear behavior.

Cherry Jell-O^®^ samples exhibited strain-stiffening (e_3_/e_1_ > 0) intracycle behavior in the nonlinear region. Based on the definition proposed by Ewoldt and Bharadwaj [30], large strain rates were the driving force behind this nonlinear elastic response, indicating that the network response was more sensitive to deformation rate than to deformation amplitude. Beyond the crossover point (γ_0_: 160%), a pronounced intracycle strain-stiffening response became dominant, likely due to the presence of helix-based junction zones in gelatin networks [17]. On the other hand, shear-thickening (v_3_/v_1_ > 0) intracycle behavior was observed in the nonlinear region. The origin of shear-thinning behavior lies in the orientation of polymer chains or the alignment of microstructures along the flow direction [26]. The magnitude of v_3_/v_1_ decreased to values below 0 at the maximum strain in the test (γ_0_: 400%), indicating a transition to intracycle shear-thinning behavior (v_3_/v_1_ < 0) at very high strain.

Strain-stiffening behavior was also evident in alginate–xanthan gum hydrogels crosslinked with calcium [31], polysaccharide of *Ficus pumila* and wheat starch composite gels [28], and hemp protein isolate-based hydrogels [32]. In addition, these gels also exhibited a transition from shear-thickening to shear-thinning behavior with increasing deformation. The nonlinear shear-thickening behavior has been attributed to microstructural changes induced by large deformations [26]. Oyinloye and Yoon [33] reported a shift from strain-stiffening to strain-softening behavior in fish myofibrillar protein gels, due to the weakening of the internal bonding structure as strain increased. According to the study reported by Hilliou et al. [34], the LAOS behavior of hybrid carrageenan gels varied depending on the concentration of iota-carrageenan gum, and gels with iota-carrageenan content below 30 mol.% exhibited strain-softening behavior. This behavior was attributed to the formation of a denser, more crosslinked network structure that restricts the strain-induced stretching of structural elements. Overall, whether a hydrogel exhibits intracycle strain stiffening/softening or shear thickening/thinning behavior appears to depend strongly on its molecular composition and network structure. The LAOS parameters of cherry Jell-O^®^ revealed that both the elastic and viscous components were affected by increasing strain amplitudes, with a more pronounced effect in the elastic stress response observed beyond γ_0_: 100%. This is due to the stable structure of Jell-O^®^ primarily formed by gelatin, the presence of bound water associated with the gelatin network [35], and the stabilized pH conditions maintained by buffering agents [7], all of which contribute to maintaining the integrity of the gel network under increased strain.

#### 2.2.3. The Elastic Lissajous–Bowditch Curves

**Figure 4:**
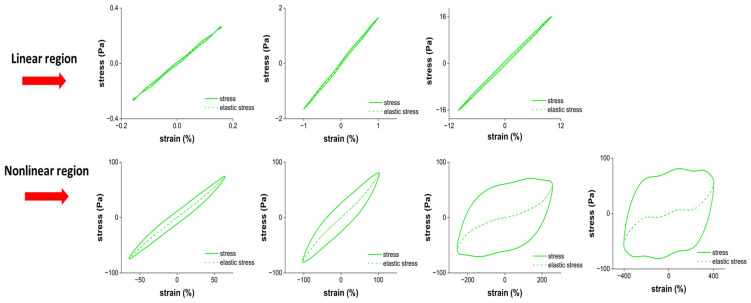
Elastic Lissajous curves of cherry Jell-O^®^.

Figure 4 shows the elastic Lissajous–Bowditch curves. They are plots of the intracycle stress–strain response of the sample as a function of increasing strain amplitudes. The elastic Lissajous–Bowditch curves for Jell-O^®^ showed a straight line in the linear region as expected, indicating a predominantly elastic response. As the strain amplitude increased above γ_0_: 12%, the curves progressively widened as a result of the nonlinearity in the stress–strain behavior. The increased widening of the elliptical trajectories in the LAOS region (beyond γ_0_ ≥ 50%) showed elastic softening [36]. This finding is consistent with the decay in G′ observed at around 100% strain (Figure 1a). At strain amplitudes beyond 100%, the elastic Lissajous curves deviated from their elliptical shape and ballooned into a rectangular form indicative of predominantly viscous behavior. This finding is consistent with the increase in tanδ values above 1 beyond γ_0_: 200%, as shown in Figure 1b. Besides broadening, the elastic Lissajous curves also exhibited distorted shapes in the non-linear region (Figure 4). Such distortions indicate intra-cycle strain-stiffening behavior [37]. This result is consistent with the e_3_/e_1_ values shown in Figure 3. The distortions at high strains indicate microstructural changes, including disruption of the protein network and the rearrangement of protein aggregates [33]. This rearrangement is mainly attributed to the ability of hydrogen bonds to interchange under the applied deformations and weakening of hydrophobic interactions within the gelatin network. leading to partial unwinding of triple helices and reorganization of aggregates [38,39].

#### 2.2.4. The Viscous Lissajous–Bowditch Curves

**Figure 5:**
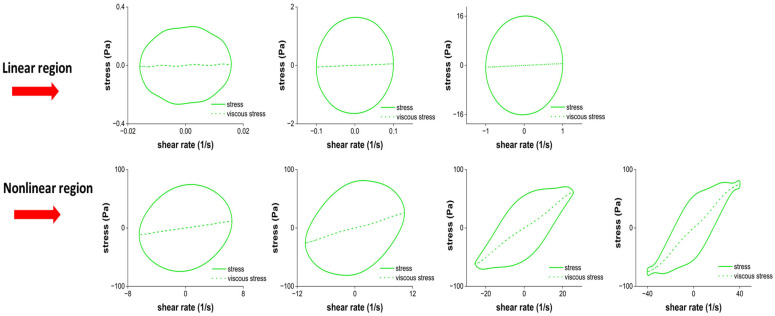
Viscous Lissajous curves of cherry Jell-O^®^.

The sample exhibited circular Lissajous shapes (Figure 5) up to γ̇: 12 s^−1^, with the most pronounced circular response at 1 s^−1^, indicating a predominantly viscous behavior. Beyond this point, loops became increasingly distorted and exhibited shear-thinning behavior. Similar behavior has been observed in other protein-based systems, including pea protein isolate particle gels [40], soy protein gels [41], and κ-carrageenan/walnut protein emulsion gels [42]. Considering the overall structural response at larger deformations, the samples underwent a rapid intra-cycle transition from an elastically stiffening elastoplastic material to a shear-thinning fluid-like material [17].

The results obtained through the elastic and viscous Lissajous–Bowditch curves collectively revealed the progressive change in viscoelasticity of the Jell-O^®^ samples when exposed to increasing strain amplitudes in the nonlinear region.

### 2.3. FTIR Spectroscopy

The results of SDS PAGE are shown in the Supplementary Materials, and it was found that the average molecular weight of the protein was 179.27 kDa, leading to an average chain length of 1630 amino acids, calculated from the equation given in Section 4.4. This value was then used in the estimation of the count of residues involved in the formation of secondary structures.

**Figure 6:**
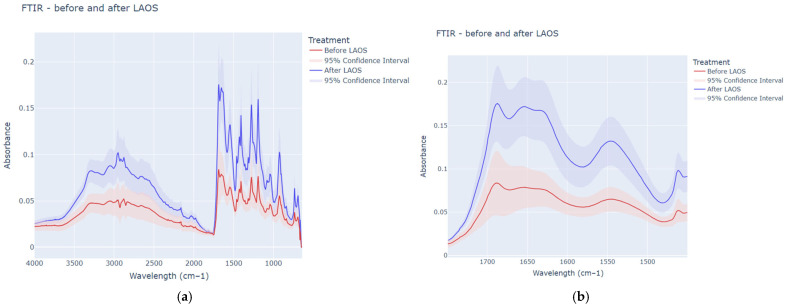
The FTIR spectra of the cherry Jell-O^®^ from before and after the LAOS deformation (**a**) and the Amide I and Amide II regions of the spectra (**b**).

The FTIR spectra of the cherry Jell-O^®^ from before and after the LAOS deformation are shown in Figure 6. These figures show the averaged FTIR spectra after baseline correction using OMNIC’s auto-baseline function.

FTIR spectra showed many characteristic peaks associated with water, proteins, and various other materials (sodium citrate, aspartame, etc.) that exist in the impure gelatin. The peaks in the region from 3500 to 3000 cm^−1^ are associated with O-H stretch in water molecules, and this region can be used to gain insight into the relative proportions of free and bound water in the sample [43,44]. Figure 6a shows that the peak at ~3290 cm^−1^ increased after LAOS deformation, indicating a higher proportion of free water in this sample [36]. When gels form, they often trap water within their helical structure [45]. The increase in peak intensity after LAOS suggested that the breakdown of the protein network altered the distribution of water throughout the gel network, leading to an increase in free water.

Observing the Amide I and Amide II regions of the spectra (Figure 6b), an increase in peak intensity is observed after the LAOS deformation treatment. This is surprising, as it would suggest an increase in the structuring of the protein, which is contrary to what would be expected after a large shear force is applied to a material. However, this data is supported by the secondary structure and hydrogen bonding analysis (Table 1). This observation can be rationalized by considering that the Amide I region is representative of secondary structure due to differences in the carbonyl stretch of the protein backbone [46], therefore it is an indicator of intramolecular interactions within the protein, not intermolecular interactions between protein chains. In the LAOS treatment, the intermolecular hydrogen bonds that define the gel network are disrupted by the large shear forces applied [47], leaving several unsatisfied hydrogen bonding sites on the protein backbone. When this occurs, a thermodynamic force drives the reformation of hydrogen bonds to minimize the free energy in the system [48]. A similar trend was observed in thermoset pea protein gels prepared using pea protein isolate that had been pre-treated with ethanol, shear, or low temperatures, showing that the gelled materials increased in ordered secondary structure (β-Sheets, α-helices) after gelation [49], which also increased the count of hydrogen bonds in the gels [50].

The secondary structure results in Table 1 indicated an increase in helix content, suggesting that, in an effort to minimize the free energy in the system, the protein adopted a more helical conformation, reforming some intramolecular hydrogen bonds as a result of LAOS deformation. It is possible this occurred because, after the breakdown of intermolecular interactions form high shear forces, the closest reactive sites to reform hydrogen bonds were within the protein, not with adjacent chains that were still moving from the applied shear forces. This would limit the reformation of intermolecular interactions and apparently promotes intramolecular interactions, thereby leading to more liquid-like behavior in the system due to non-interacting protein strands. A similar relationship was observed in thermoset pea protein gels, where an increase in the count of hydrogen bonds derived from secondary structure was correlated with a decrease in the G′ of the samples studied [40].

While not the explicit focus of this work, several differences were observed in the fingerprint region of the FTIR spectra as well, as shown in Supplementary Materials Figure S1. This region is defined by carbohydrates and minor ingredients (acidulants, buffers, etc.) in the Jell-O^®^. A new peak is formed at ~1335 cm^−1^ in the LAOS-treated samples, along with an increase in the peak at 1355 cm^−1^ both of which are peaks that also appear in a reference spectra for aspartame or maltodextrin, and could indicate differences in the way these ingredients are incorporated into the gel network [51,52].

There is also a new shoulder formed at ~1247 cm^−1^ possibly due to a change in the interaction of disodium phosphate [53], along with a change in the peak shape at 1079 cm^−1^ (disodium phosphate) and 1043 cm^−1^ (aspartame). These collectively suggested that while the LAOS treatment meaningfully altered the protein network, many changes and new interactions occurred within the other components of the Jell-O^®^ as well.

### 2.4. Effect of LAOS Deformation on the Frequency-Dependent Viscoelastic Properties of Cherry Jell-O^®^

**Figure 7:**
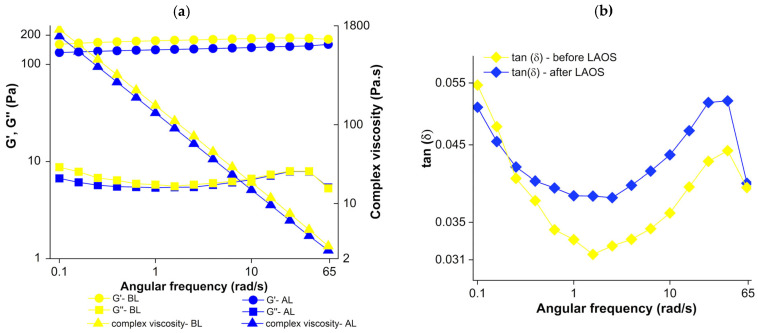
Frequency sweep results for cherry Jell-O^®^ before and after LAOS deformation. (**a**) Storage modulus (G′), loss modulus (G″), and complex viscosity as a function of angular frequency (rad/s); (**b**) tanδ versus as a function of angular frequency (rad/s).

The effect of LAOS deformation on the frequency-dependent viscoelastic properties of cherry Jell-O^®^ samples is shown in Figure 7a (G′, G″, and complex viscosity) and Figure 7b (tanδ). The complex viscosity decreased with increasing frequency, indicating that the samples exhibited characteristic shear-thinning behavior. This behavior can be attributed to the disentanglement of long-chain polymers triggered by high frequencies, leading to a decrease in complex viscosity [54]. After LAOS deformation, the complex viscosity decreased significantly (*p* < 0.05) (Figure 7a); however, at the highest frequencies (40 rad/s and 65 rad/s), this decrease was not significant (*p* > 0.05). This may be explained by the short polymer network rearrangement time at higher frequencies [55], which limits the viscous contribution and reduces the viscosity differences between the samples.

Both G′ and G″ exhibited weak frequency dependence. Such behavior has been reported for various gels in the literature [56,57]. G′ was an order of magnitude higher than G″, indicating the sample exhibited strong elastic behavior. G″ remained almost constant even after LAOS deformation, except at the lowest frequencies. In contrast, G′ was slightly higher before LAOS deformation compared to after LAOS deformation throughout the frequency sweep (Figure 7a). These results suggested that the effect of LAOS deformation had a greater effect on G′ compared to G″, and exposure to LAOS deformation resulted in a decrease in elasticity. This finding is consistent with the increase in tanδ values after LAOS deformation observed in Figure 7b. This increase was significant (*p* < 0.05) within the moderate frequency range (from 0.6 rad/s to 25 rad/s). However, tanδ values remained below 1 even after LAOS deformation, indicating that the samples continued to exhibit predominantly elastic behavior. This phenomenon can be explained by the reorganization of the protein network upon removal of LAOS deformation, resulting in structural recovery and a decrease in tanδ values. This finding was also consistent with Table 1, which indicates an increase in α-helix content. These results showed that although LAOS deformation induced microstructural changes in the cherry Jell-O^®^ samples, they maintained their elastic structure thanks to a strong and resilient network structure.

## 3. Conclusions

Cherry Jell-O^®^ maintained solid-like behavior even under high deformations (up to γ_0_: 160%), indicating its predominantly elastic character. Chebyshev coefficients revealed that intracycle strain-stiffening and intracycle shear-thickening behavior were especially evident in the LAOS region. Throughout the high-strain regime (γ_0_ > 100%), increasing strain amplitudes had a more pronounced effect on the elastic stress response, showing that intracycle strain-stiffening behavior dominates the nonlinear response. A transition from intracycle shear-thickening to shear-thinning behavior was observed at the maximum strain, suggesting that high strain amplitudes facilitated material flow.

In the LAOS region, distorted elliptical shapes were observed, suggesting that the sample structure was disrupted, leading to a loss of elasticity. The viscous Lissajous–Bowditch curves indicated shear-thinning behavior with increasing LAOS deformation. Exposure to LAOS deformation decreased elasticity and resulted in an associated increase in tanδ values. FTIR results showed that secondary structure and intramolecular hydrogen bonds were affected significantly after LAOS deformation, except for the β-sheet structure. These findings suggested that while LAOS deformation disrupted intermolecular hydrogen bonds causing shifts in intramolecular hydrogen bonds, the preservation of β-sheet structures provides a stable backbone that maintains the integrity of the gel network. The stop-flow LAOS-frequency sweep approach provided insight into how LAOS deformation influences the microstructure and revealed that the protein network reorganizes after the removal of deformation. Nonlinear viscoelastic characteristics are directly correlated to tribology and textural properties; therefore, they define consumer acceptability. Ultimately, the findings of this study can provide guidance on optimizing key gel parameters, as well as form a foundation for the development of gel-based food products, with particular emphasis on rheological properties.

## 4. Materials and Methods

### 4.1. Material

Zero-sugar cherry Jell-O^®^ samples (ready-to-eat gel form) were purchased from Walmart in West Lafayette, IN, USA. The samples were kept refrigerated at 4 °C until analysis.

### 4.2. Rheological Measurements

Rheological measurements were performed using a stress-controlled Discovery Hybrid Rheometer (DHR-3, TA-Instruments, New Castle, DE, USA). All rheological measurements were conducted at 20 °C using an X-hatch parallel plate geometry (20 mm diameter, 1.5 mm gap), over a strain range of 0.01–400% at a constant angular frequency of 10 rad/s. The sample was allowed to rest until the axial force was below 1 N prior to analysis. To determine MAOS boundaries, the method reported by Erturk et al. [22] was used. The ratio of the third (3rd) harmonic intensity to the first (1st) harmonic intensity was calculated, and this ratio was plotted as a function of strain (%). The boundaries of the MAOS region were determined based on the systematic increase in the I_3_/I_1_ ratio [22]. The subsequent step in the MAOS analysis involved examining the normalized G′ and G″ data. For this purpose, the G′ and G″ moduli obtained across the strain range of 0.01–400% were normalized by the G′ and G″ values from the SAOS region (at γ_0_: 1%) and plotted against the strain amplitude on a log-log scale [58].

To assess the changes in the viscoelastic properties of the samples induced by large deformations, frequency sweeps (0.1–65 rad/s at constant strain of 1%) were performed in the linear viscoelastic region both before and after the LAOS tests. All rheological data were analyzed using the TRIOS software version 5.1.1.46572 (TA Instruments, Schaumburg, IL, USA) and visualized using OriginPro 2023-b.

### 4.3. Sodium Dodecyl Sulfate–Polyacrylamide Gel Electrophoresis (SDS-PAGE)

The average molecular weight of the protein was estimated using gel densitometry, following the method of Bonilla et al. [59]. This weight was used in the estimation of hydrogen bonds, detailed below. The gel was analyzed in ImageJ (https://imagej.net/ij/ accessed on 15 March 2026, National Institutes of Health, Bethesda, MD, USA) using the analyze gel tool. The lane on the SDS-PAGE gel containing the Jell-O sample was plotted, and the areas associated with the protein bands were integrated to find the area associated with each molecular weight fraction in the protein. A weighted average was taken of the different protein bands to determine the average molecular weight of the protein in Jell-O^®^.

### 4.4. Fourier Transform Infrared Spectroscopy (FTIR)

Jell-O^®^ slices were cut to serve as a control sample from before LAOS deformation. A second slice was cut and placed on the stage of the rheometer and treated as described above. The slices from before and after the LAOS deformation treatment were freeze-dried and characterized using an FTIR spectrometer (ThermoNicolet 6700, Thermo Fisher Scientific, Madison, WI, USA) in the wavenumber range of 400–6000 cm^−1^ and 64 scans per sample. FTIR was used to probe the molecular structure of the sample before and after deformation. These measurements were conducted in triplicate and exported as .csv files for visualizations prepared using Plotly in Python version 3.9 [60].

To estimate the secondary structure of the protein from the FTIR spectra, the spectra were treated using tools available in the OMNIC software version 8.3 (Thermo Fisher Scientific, Waltham, MA, USA) following the method described in [60]. First, samples were baseline corrected using the auto baseline tool, followed by applying the Fourier Self-Deconvolution tool. Using the deconvoluted spectra, Gaussian curves were fit to the original spectra using a constant baseline in OMNIC. These curves were integrated, and the area of each peak was divided by the sum of all areas to assign the percentage of a secondary structure. Peaks between ~1600–1605 cm^−1^ and ~1624–1626 cm^−1^ were defined as β-sheets, 1655–1657 cm^−1^ were defined as α-helices, and 1669–1691 cm^−1^ were assigned as β-turns [46,61].

Protein secondary structure is stabilized by hydrogen bonds, with one hydrogen bond every 3.6 amino acids in α-helices or every other amino acid in antiparallel β-sheets [62]. Given this relationship, previous work utilized X-ray crystallography structures deposited in the Protein Data Bank from 600 proteins to train a least squared regression model that predicts the count of hydrogen bonds in a protein using the secondary structure as inputs to the model, and the model was validated on a separate database of 560 proteins [49]. The development of this linear model allows for an estimation of the count of hydrogen bonds in a protein material as a function of the secondary structure of the protein with the following equation:

Intramolecular hBonds = −7.85 + 1.05 × (helix residues) + 1.11 × (sheet residues) + 0.65 × (coil residues)

Since the model was developed using bioinformatic models that had the count of residues involved in the formation of secondary structures as inputs, there was a need to convert the percentages of secondary structure from FTIR to an estimate of the count of residues involved in the formation of a secondary structure. This was done following the method recommended in [49]. While all amino acids have different molecular weights, the average molecular weight of an amino acid is 110 Da. Therefore, given the molecular weight of a protein (obtained from SDS-PAGE in this work), it is possible to use the percentage of secondary structures to estimate the count of residues as follows:

Count of residues in Secondary Structure = secondary structure (%) × (Average Chain Length)

Average Chain Length = Average Molecular Weight (Da)/110 (Da/amino acid)

In the above, the average chain length is the count of amino acids in an average protein chain in the material, and the count of residues is the number of residues involved in the formation of a given secondary structure, i.e., α-helices. These values were used as inputs into the Intramolecular hBonds equation to estimate the count of intramolecular hydrogen bonds in the protein. Since the model was based on protein structures, this approach can be used to estimate the count of intramolecular hydrogen bonds in the protein, but it is not reflective of intermolecular hydrogen bonds between protein chains, as may form in a gel system.

### 4.5. Statistical Analysis

All rheological experiments were conducted at least three times, and a paired-samples *t*-test was employed to compare the frequency-dependent viscoelastic properties before and after LAOS deformation, along with the differences in secondary structure before and after deformation, with a significance level of *p* < 0.05.

## Figures and Tables

**Table 1:** Secondary structure and intramolecular hydrogen bonding analysis.

	Before LAOS	After LAOS
Sheet	40.68 ± 2.04 ^a^	40.95 ± 0.71 ^a^
Helix	16.09 ± 2.53 ^a^	33.11 ± 3.17 ^b^
Turn	43.23 ± 0.48 ^a^	25.94 ± 3.89 ^b^
Intramolecular hBonds	82.30 ± 0.07 ^a^	89.23 ± 1.60 ^b^

Note: Values are presented as mean ± standard deviation. Significant differences within the same row were evaluated. Means that do not share a letter are significantly different (*p* < 0.05).

## Data Availability

The data supporting the findings of this study are available from the corresponding author upon reasonable request.

## Supplementary Materials

The following supporting information can be downloaded at: https://www.mdpi.com/article/10.3390/gels12040295/s1, Figure S1: Fingerprint region of Jell-O^®^ samples in the FTIR spectra; Figure S2: SDS-PAGE pattern of Cherry Jell-O^®^.

## Nomenclature/Abbreviations

The following nomenclature/abbreviations are used in this manuscript:

Rheology Nomenclature	
γ_0_	strain amplitude (−)
γ_cri_	critical strain (−)
G′	elastic (storage) modulus (Pa)
G″	viscous (loss) modulus (Pa)
tanδ	loss tangent (−), where δ is the phase angle (rad)
ω	angular frequency (rad/s)
γ̇:	shear rate (s^−1^)
I_3_	third harmonic intensity
I_1_	first harmonic intensity
e_3_/e_1_	elastic Chebyshev coefficient
v_3_/v_1_	viscous Chebyshev coefficient
Abbreviations	
SAOS	Small amplitude oscillatory shear
MAOS	Medium amplitude oscillatory shear
LAOS	Large amplitude oscillatory shear
HPMC	Hydroxypropyl methylcellulose
HPMCP	Hydroxypropyl methylcellulose phthalate
