# Supplementary figures and images for: Probing the Small, Medium and Large Amplitude Rheological Properties of Cherry Jell-O® as a Model System for Edible Gels
Source: Gels. 2026 Apr 1;12(4):295. doi: 10.3390/gels12040295 (PMC13116617; doi:10.3390/gels12040295)

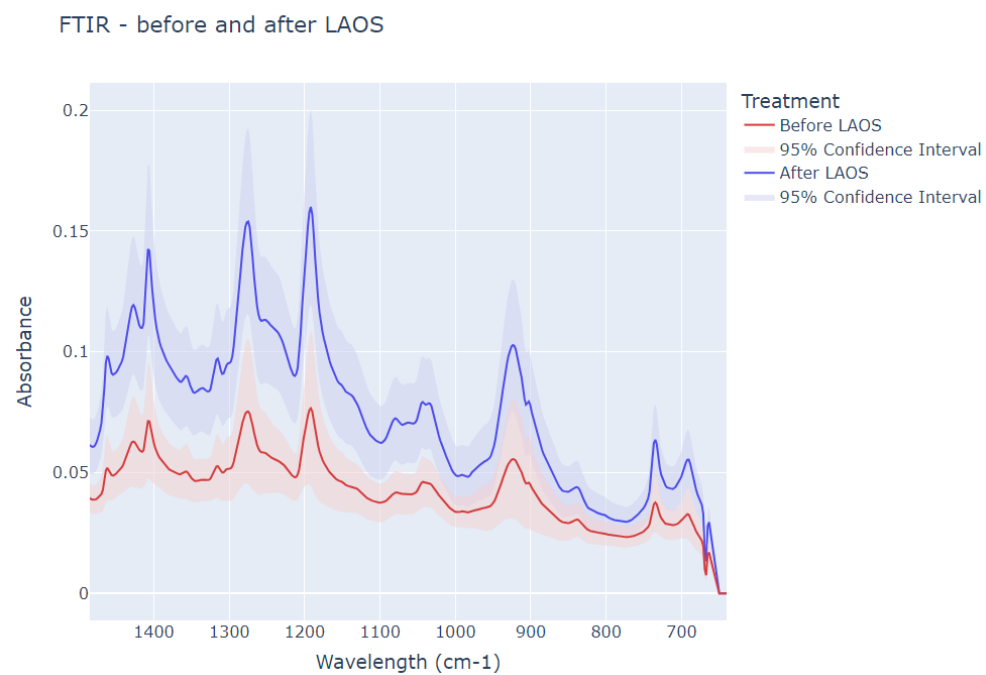

**Figure S1.** Fingerprint region of Jell-O® samples in the FTIR spectra

Supplement: Supplementary file 1 [file gels-12-00295-s001.zip › Figure S1.pdf]

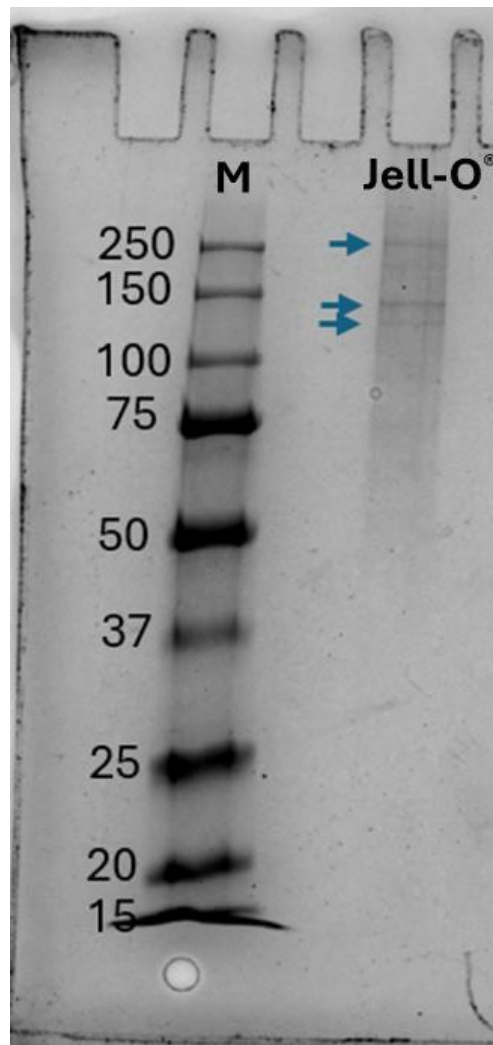

**Figure S2.** SDS-PAGE pattern of Cherry Jell-O®

Supplement: Supplementary file 1 [file gels-12-00295-s001.zip › Figure S2.pdf]
